# Impact of Two Strains of *Rhizobium leguminosarum* on the Adaptation to Terminal Water Deficit of Two Cultivars *Vicia faba*

**DOI:** 10.3390/plants11040515

**Published:** 2022-02-14

**Authors:** Ihsein Rokia Amine-Khodja, Alexandre Boscari, Nassira Riah, Maya Kechid, Rim Tinhinen Maougal, Nadir Belbekri, Abdelhamid Djekoun

**Affiliations:** 1 Genetic, Biochemistry and Plant Biotechnology Laboratory, Faculty of Natural Sciences, Constantine 1 University, Route Ain El Bey, Constantine 25000, Algeria; ihsenerokia@gmail.com (I.R.A.-K.); riah_nassira@umc.edu.dz (N.R.); mkechid23@yahoo.fr (M.K.); rym.maougal@umc.edu.dz (R.T.M.); nbelbekri@hotmail.com (N.B.); djak2591@gmail.com (A.D.); 2 INRAE, Université Côte d’Azur, CNRS, ISA, 06903 Sophia Antipolis, France

**Keywords:** faba bean, *Rhizobium* strains, drought stress, symbiosis, antioxidants, osmoprotectants

## Abstract

Drought stress has become one of the most uncontrolled and unpredictable constraints on crop production. The purpose of this study was to evaluate the impacts of two different *Rhizobium leguminosarum* strains on terminal drought tolerance induction in two faba bean genotypes cultivated in Algeria, Aquadulce and Maltais. To this end, we measured physiological parameters—osmoprotectants accumulation, oxidative stress markers and enzyme activities—to assess the effect of *R. leguminosarum* inoculation on *V. faba* under terminal water deficiency conditions in greenhouse trials. Upregulation of anti-oxidative mechanisms and production of compatible solutes were found differentially activated according to *Rhizobium* strain. Drought stress resilience of the Maltais variety was improved using the local *Rhizobium* strain OL13 compared to the common strain 3841. Symbiosis with OL13 strain leads in particular to a much better production of proline and soluble sugar in nodules but also in roots and leaves of Maltais plant. Even if additional work is still necessary to decipher the mechanism by which a *Rhizobium* strain can affect the accumulation of osmoprotectants or cellular redox status in all the plants, inoculation with selected *Rhizobium* could be a promising strategy for improving water stress management in the forthcoming era of climate change.

## 1. Introduction

Legumes are an important source of protein suitable for livestock feed and human consumption [1]. Additionally, their ability to establish a symbiosis with specific *Rhizobium* bacteria that enzymatically convert atmospheric nitrogen into organic form in the host roots, provides the benefit of reduced fertilizer input and enhanced soil biological activity which helps in improving and sustaining the soil productivity [2,3]. Faba bean (*Vicia faba* L.) is one of the most important legume crops globally [2], owing to its high protein content (up to 35% in dry seeds), carbohydrates, fiber, and vitamins [1,4]. Recently, interest in this crop has increased, but it is reputed to be relatively more sensitive to drought than other grain legumes, including common bean, pea and chickpea [5,6,7]. Due to global climate change, drought has become one of the most uncontrolled and unpredictable constraints, with adverse effects on crop production worldwide [8,9], and affecting roughly 64% of the global land area [10].

The susceptibility of legumes to drought depends on many factors such as the growth stage of the plant, genetic potential, duration and severity of the stress [11]. Although drought hampers the productivity of grain legumes at all growth stages, in the Mediterranean-type climates, where the rainfall is inadequate and erratically distributed during the year, the faba bean often faces drought at the terminal stages during reproductive and grain development [5,12,13]. Indeed, faba bean are strongly sensitive to drought during flowering, early podding, and grain filling stages [2,14,15], but the early reproductive phase is the most sensitive stage [16,17]. However, large differences in drought tolerance are observed amongst the faba bean varieties [18,19,20].

Undeniably, drought stress induces a significant reduction in plant growth parameters [21], shoot and root biomass [22]. Drought affects many aspects of plant physiology, decreasing photosynthesis rates, chlorophyll and carotenoids content, stomatal conductance, and disturbs plant water relations leading to a reduction in plant growth and productivity [23,24]. Furthermore, symbiotic nitrogen fixation is also particularly sensitive to drought stress [25,26]. Among various defensive mechanisms, osmotic adjustment provided by the accumulation of osmoprotectants may confer tolerance to drought injuries by maintaining high tissue water potential [27,28]. However, in faba beans, drought tolerance through osmotic adjustment has not yet been demonstrated in the wide germplasm [13]. In addition, due to drought stress, whole plant metabolism is dramatically affected by the over-production of reactive oxygen species (ROS) that are responsible for oxidation of multicellular components like proteins, lipids, DNA and RNA, leading to cell necrosis and cell death [29]. With evolution, plants have adjusted to the changing environments and have learnt ways to counter the lethal effects of ROS through various enzymatic and non-enzymatic antioxidants which operate in different cellular organelles to scavenge the ROS [30]. Among the important ROS-scavenging enzymes we find superoxide dismutase, peroxidases, and catalase [31].

Keeping in mind the importance of this crop for humans as well as animals and the environment, the purpose of this study was to evaluate the influence of *Rhizobium leguminosarum* strains on terminal drought tolerance of two faba bean genotypes grown in Algeria. To this end, we measured physiological parameters—osmoprotectants accumulation, oxidative stress markers and enzymes activities—in leaves, roots and nodules from two genotypes of *V. faba*—Aquadulce (AQD) and Maltais (MLT)—inoculated with a standard *R. leguminosarum bv. viciae* 3841 strain or with the local strain OL13 isolated from the nodule of *Lens culinaris* from a semi-arid Algerian region [32] and submitted or not to water deficiency furing their terminal growth stages in greenhouse trials. Understanding the impact of *R. Legumunisarum* strain on physiological and biochemical properties measured on plant partners is important, in particular to identify symbiotic couples that are more efficient under these conditions of abiotic stress.

## 2. Results

### 2.1. Growth Parameters Analyses on Vicia faba with Different Rhizobium leguminosarum Strains under Drought Stress

**Figure 1:**
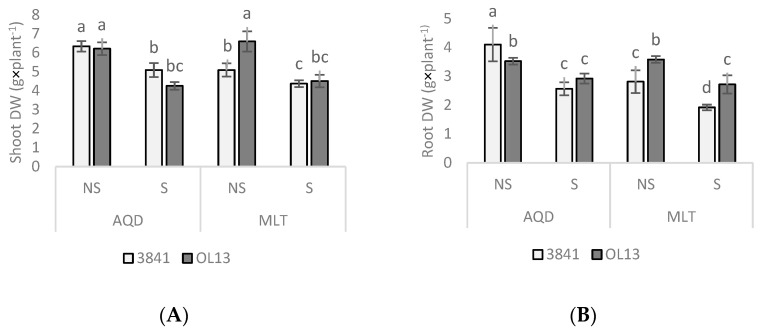
Dry matter yield responses to water deficiency. Seventy day-old *Vicia faba* plants, previously individually inoculated with *Rhizobium* strains (3841-light gray bars) and (OL13-dark gray bars), were grown under control conditions (Noted: NS) or under water limited conditions (Noted: S). (**A**) Shoot, (**B**) root plant dry weights (DWs) were measured after seventy day of growth. AQD: Aquadulce; MLT: Maltais. The values shown are the mean ± SD of three independent replicates. Differences in the data were considered significantly different at the 0.05 level of probability by Student-Newman-Keuls Test (indicated by different letters).

Growth performances of two different faba bean genotypes coupled individually with two different *R. leguminosarum* strains were analyzed under well-watered and limited-water conditions applied at the terminal growth stage, during the reproductive and grain development stages, a critical stage in legume grains. Inhibition of growth, which results in reduced dry matter yield, is one of the most common symptoms of dehydration [33,34]. Under water deficit conditions, shoot and root DW were significantly reduced compared to those achieved under control conditions (Figure 1). For shoot DW, the AQD genotype showed the same biomass for both studied strains and this for both watering conditions. In contrast, MLT genotype recorded a higher shoot DW when inoculated with OL13 under well-watered conditions. For root DW, under well-watered conditions, AQD present a higher biomass when inoculated with 3841 stain than with OL13 strain. Under drought conditions, AQD did not show any difference between the two strains (Figure 1). However, MLT root DWs were significantly enhanced by the OL13 strain compared to 3841 strain. These results show that growth parameters were significantly affected by the *Rhizobium* strain in the two considered organs for both genotypes, under well-water and water-limited conditions. OL13 strain produces a net improvement of root DW in the MLT genotype under both watering conditions.

### 2.2. Stomatal Conductance and Photosynthetic Pigments Responses to Water-Limited Conditions

Faba bean responses to water deficit were analyzed by measuring select physiological and biochemical markers. Stomatal conductance is considered as an important physiological marker for screening faba bean genotypes under water deficit conditions. As biochemical markers, chlorophyll (Chl) and carotenoid (Car) contents were measured.

**Figure 2:**
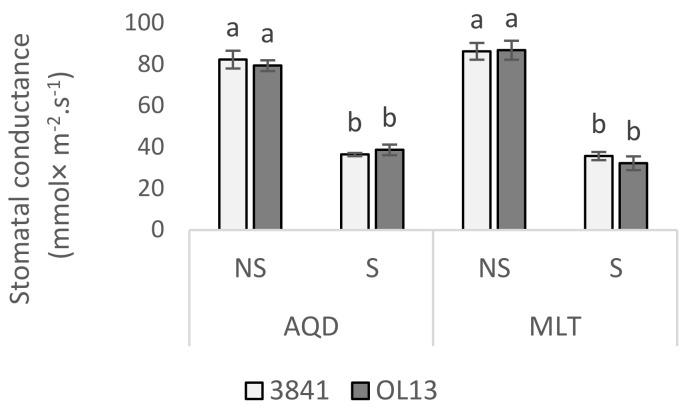
Stomatal conductance influenced by water deficit conditions. Seventy day-old *Vicia faba* plants, previously individually inoculated with *Rhizobium* strains (3841-light gray bars) and (OL13-dark gray bars), were grown under control conditions (Noted: NS) or under water limited conditions (Noted: S). AQD: Aquadulce; MLT: Maltais. The values shown are the mean ± SD of three independent replicates. Differences in the data were considered significantly different at the 0.05 level of probability by Student-Newman-Keuls Test (indicated by different letters).

Stomatal conductance was not significantly different between the two cultivars and between the different *Rhizobium* inoculations under both watering conditions (Figure 2). Under limited-water conditions, the stomatal conductance decreased for both genotypes and both bacterial treatments. 

**Figure 3:**
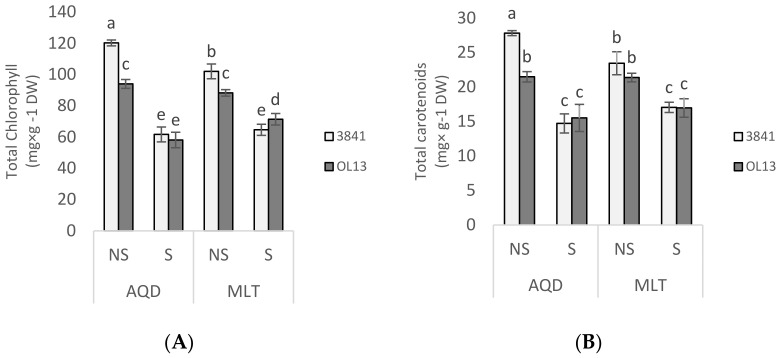
Photosynthetic pigments content in leaves. Seventy day-old *Vicia faba* plants, previously individually inoculated with *Rhizobium* strains (3841-light gray bars) and (OL13-dark gray bars), were grown under control conditions (Noted: NS) or under water limited conditions (Noted: S). (**A**) Total Chlorophyll, (**B**) Total carotenoids. AQD: Aquadulce; MLT: Maltais. The values shown are the mean ± SD of three independent replicates. Differences in the data were considered significantly different at the 0.05 level of probability by Student-Newman-Keuls Test (indicated by different letters).

Total Chl and Car contents were significantly reduced under water limited conditions (Figure 3). Under well-watered conditions, a higher content of Chl was measured for both genotype inoculated with 3841 compared to OL13. However, no significant changes were recorded between the two strains for both studied genotypes under limited water conditions. For Car content, the higher value was recorded for AQD inoculated with the strain 3841, however no significant differences were registered for MLT.

### 2.3. Accumulation of Osmoprotectants in Response to Water Deficit

**Figure 4:**
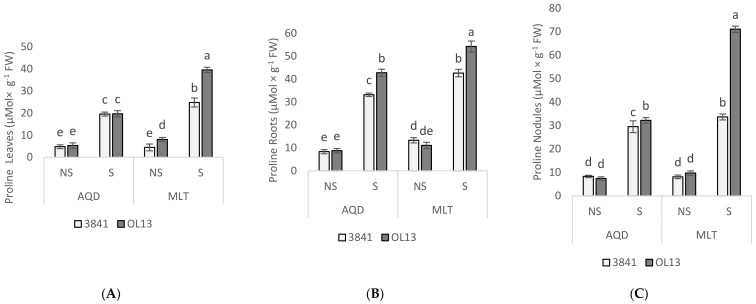
Proline content in leaves, roots and nodules. Seventy day-old *Vicia faba* plants, previously individually inoculated with *Rhizobium* strains (3841-light gray bars) and (OL13-dark gray bars), were grown under control conditions (Noted: NS) or under water limited conditions (Noted: S). (**A**) Leaves, (**B**) Roots and (**C**) Nodules. AQD: Aquadulce; MLT: Maltais. The values shown are the mean ± SD of three independent replicates. Differences in the data were considered significantly different at the 0.05 level of probability by Student-Newman-Keuls Test (indicated by different letters).

**Figure 5:**
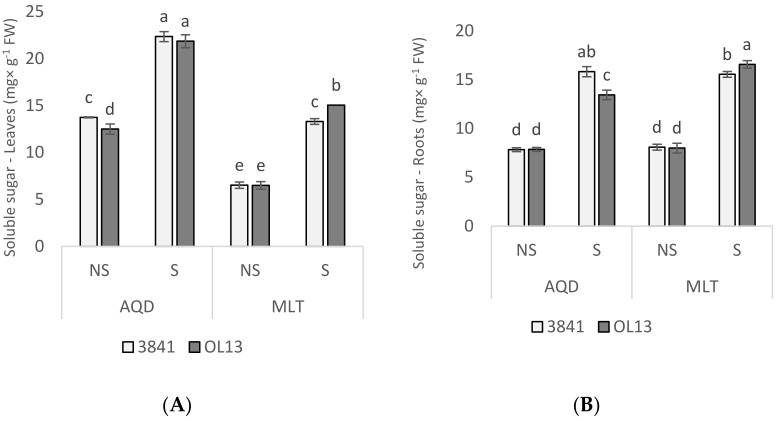
Soluble sugar content in leaves and roots. Seventy day-old *Vicia faba* plants, previously individually inoculated with *Rhizobium* strains (3841-light gray bars) and (OL13-dark gray bars), were grown under control conditions (Noted: NS) or under water limited conditions (Noted: S). (**A**) Leaves and (**B**) Roots. AQD: Aquadulce; MLT: Maltais. The values shown are the mean ± SD of three independent replicates. Differences in the data were considered significantly different at the 0.05 level of probability by Student-Newman-Keuls Test (indicated by different letters).

Proline and soluble sugar are important biochemical indicators of stress tolerance in plants. Their accumulation by plant tissues under water-limited conditions is an adaptive response [33,35]. We measured the content of proline in leaves, roots and nodules (Figure 4) and the soluble sugar content in leaves and roots (Figure 5). Proline contents were not significantly different in these organs of the two inoculated plants under well-watered conditions. Under water deficit condition, proline content strongly increased in all organs of the two genotypes (Figure 5). The highest accumulations of proline were observed in roots and nodules for both genotypes. The highest accumulations were observed in MLT inoculated by OL13 with 391%, 390% and 630% increases in leaves, roots and nodules, respectively. MLT nodules from strain OL13 show practically double the proline content compared to MLT nodules from strain 3841 and AQD nodules under the same water stress conditions.

Accumulation of soluble sugars as osmolytes is another method of acclimatisation towards osmotic adjustment under drought stress [36] and was also investigated in leaves and roots of the two studied genotypes. In our study, a significant difference was observed in the accumulation of sugar in plants under water deficit conditions in both inoculated plants when compared with well-watered conditions (Figure 5A,B). In the leaves, intense soluble sugar accumulation was observed under drought stress conditions in both genotypes, being significantly higher in AQD than in MLT genotype. However, both studied *Rhizobium* strains enhanced the sugar content in AQD genotype, when the OL13 showed a significantly more sugar accumulation for MLT genotype (Figure 5A).

In roots, a similar induction of soluble sugar accumulation was observed under drought conditions, with a slightly higher accumulation in MLT with OL13 strain compared to 3841 strain and a reverse response in AQD genotype (Figure 5B). These results suggest that under the experimental conditions used sugar metabolites contribute significantly to the maintenance of osmotic potential in the shoot and roots under drought stress conditions, with a difference between the two studied genotypes and the two studied strains.

### 2.4. Measurement of Oxidative Stress Markers

Plants’ exposure to drought stress causes an increase in cellular level of reactive oxygen species (ROS) like superoxide radical (O_2_^−^), hydroxyl radicals (OH) and hydrogen peroxide (H_2_O_2_), leading to oxidative damage to proteins, DNA and lipids [29,37,38]. The MDA and H_2_O_2_ content are determinants of oxidative stress in plants under drought stress conditions, hence we measured the content of MDA (Table 1) and H_2_O_2_ in leaves and roots (Table 2). Under well-watered conditions, both inoculated plants showed similar levels of MDA contents for both shoot and root, whereas a significant increase was recorded when plants were subjected to water deficit conditions (Table 1). Regarding MDA accumulation, almost all measurements between the two genotypes were similar for the two *Rhizobium* except for significantly lower content in leaves of AQD inoculated by OL13 strains. 

Under well-watered conditions, H_2_O_2_ content was similar in leaves and roots of both genotypes and for both studied strains (Table 2). Under water deficit conditions, H_2_O_2_ content increased significantly in leaves and roots of both studied genotypes compared to well-watered plants, independently of the *Rhizobium* strain. This increase was always significantly lower in AQD than in MLT. Even if H_2_O_2_ content was lower in AQD with 3841 strain compared to OL13 strain and in MLT with OL13 strain compared to 3841, the results were not statistically valid. Taken together, the results showed that strain 3841 or OL13 did not lead to modification of MDA and H_2_O_2_ content in AQD or MLT under water stress conditions.

### 2.5. Enzymatic Activities Responses to Water Deficit

**Figure 6:**
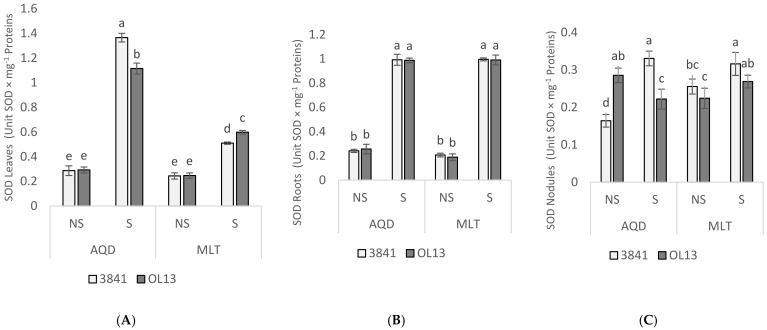
Superoxide dismutase activity in leaves, roots and nodules. Seventy day-old *Vicia faba* plants, previously individually inoculated with *Rhizobium* strains (3841-light gray bars) and (OL13-dark gray bars), were grown under control conditions (Noted: NS) or under water limited conditions (Noted: S). (**A**) Leaves, (**B**) Roots and (**C**) Nodules. AQD: Aquadulce; MLT: Maltais. The values shown are the mean ± SD of three independent replicates. Differences in the data were considered significantly different at the 0.05 level of probability by Student-Newman-Keuls Test (indicated by different letters).

**Figure 7:**
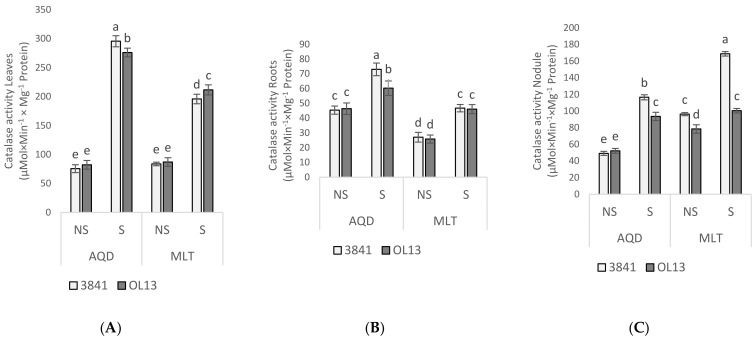
Catalase activity in leaves, roots and nodules. Seventy day-old *Vicia faba* plants, previously individually inoculated with *Rhizobium* strains (3841-light gray bars) and (OL13-dark gray bars), were grown under control conditions (Noted: NS) or under water limited conditions (Noted: S). (**A**) Leaves, (**B**) Roots and (**C**) Nodules. AQD: Aquadulce; MLT: Maltais. The values shown are the mean ± SD of three independent replicates. Differences in the data were considered significantly different at the 0.05 level of probability by Student-Newman-Keuls Test (indicated by different letters).

**Figure 8:**
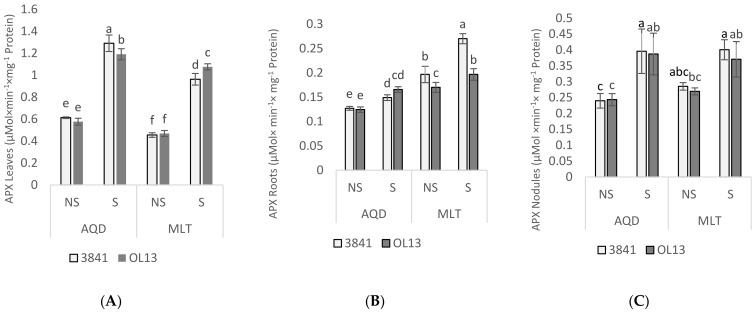
Ascorbate peroxidase activity in leaves, roots and nodules. Seventy day-old *Vicia faba* plants, previously individually inoculated with *Rhizobium* strains (3841-light gray bars) and (OL13-dark gray bars), were grown under control conditions (Noted: NS) or under water limited conditions (Noted: S). (**A**) Leaves, (**B**) Roots and (**C**) Nodules. AQD: Aquadulce; MLT: Maltais. The values shown are the mean ± SD of three independent replicates. Differences in the data were considered significantly different at the 0.05 level of probability by Student-Newman-Keuls Test (indicated by different letters).

**Figure 9:**
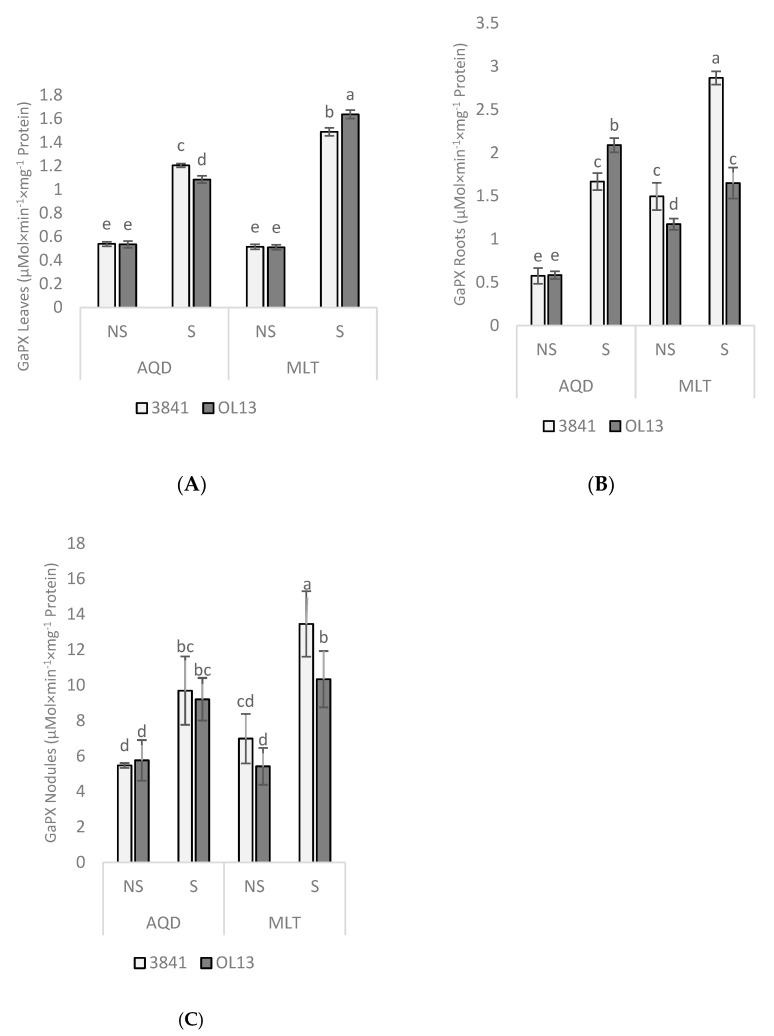
Guaiacol peroxidase activity in leaves, roots and nodules. Seventy day-old *Vicia faba* plants, previously individually inoculated with *Rhizobium* strains (3841-light gray bars) and (OL13-dark gray bars), were grown under control conditions (Noted: NS) or under water limited conditions (Noted: S). (**A**) Leaves, (**B**) Roots and (**C**) Nodules. AQD: Aquadulce; MLT: Maltais. The values shown are the mean ± SD of three independent replicates. Differences in the data were considered significantly different at the 0.05 level of probability by Student-Newman-Keuls Test (indicated by different letters).

In order to protect themselves against the effects of drought stress, plants develop antioxidant defense systems constituted by both enzymatic and non-enzymatic components [39]. The activities of the antioxidant enzymes SOD, CAT, APX and GaPX were measured as parameters of antioxidant defense in leaves, roots and nodules. SOD is a key enzyme in the regulation of intracellular concentrations of ROS; it converts superoxide radicals into H_2_O_2_, then APX, CAT, and GaPX convert H_2_O_2_ to H_2_O [40,41]. Combined action of SOD, APX, CAT, and GaPX is critical in mitigating effects of oxidative stress [42]. In the present experiment, no significant difference was recorded between the two studied strains for the leaves and roots and between the two studied genotypes under well-watered conditions (Figure 6, Figure 7, Figure 8 and Figure 9). However, activity of all antioxidant enzymes in the three-studied organs for both genotypes increased significantly under water-limited conditions.

For the leaves part, when comparing the studied enzymes activities in water-stressed condition, the AQD genotypes exhibited a significant higher activity for SOD, APX and CAT than MLT genotype. In contrast, MLT recorded a significant higher GaPX activity then AQD. Moreover, under limited-water conditions, enzymes activities in the leaves were more highly induced by 3841 strain for AQD genotype and by OL13 strain for the MLT genotype.

In the roots part, under water deficit stress, SOD activity was significantly induced by the two studied strains for both genotype, but no significant differences were recorded between the two strains (Figure 6). For CAT activity, under limited-water conditions, the only significant difference was recorded for AQD genotype inoculated by 3841 strain (Figure 7). For APX activity, under limited-water conditions, roots of MLT genotype showed the highest activity compared with AQD. The highest activity was recorded for MLT inoculated by the strain 3841, and no significant differences were recorded for AQD between inoculated plants (Figure 8). In contrast with the three previous enzymes, higher GaPX activity was measured in roots and nodules than in leaves (Figure 9). GaPX activity was more strongly enhanced by OL13 strain for AQD and by 3841 strain for MLT, thus showing a trend opposite to what was observed for the other three activities.

At the nodules level, AQD showed strong differences in function of the *Rhizobium* strain contrary to MLT. Two-fold induction of SOD activity was observed in AQD nodulated with 3841 strain submitted to drought stress. In case of MLT nodulated with OL13, high level of SOD was observed in well-watered condition with significant decrease in stress condition. Nodules from MLT genotype showed a higher catalase and GaPX activity than AQD under both watering conditions. Furthermore, this induction was stronger if inoculated with 3841 strain. A similar trend was also observed for GaPX activity. For APX activity, no significant differences between the two studied strains were recorded.

## 3. Discussion

Drought stress constraints plants and rhizobia act symbiotically to perform optimally [3]. Climate change is expected to aggravate the periods and intensity of water stress, including drought stress. Relatively little information is available concerning how drought affects the symbiotic relationship between nitrogen-fixing soil rhizobia and the host plant [43]. However, to make the symbiosis effective under water deficit conditions, legumes need to be combined with effective *Rhizobium* strains appropriate for each legume species to obtain the most effective combination [44]. In the present study, we compared AQD, a drought tolerant genotype, with a local genotype, MLT, either inoculated with a standard *R. leguminosarum bv. viciae* 3841 strain or with the local strain OL13 isolated from the nodule of *Lens culinaris* from a semi-arid Algerian region [32], for their tolerance to water deficiency during their terminal growth stages in greenhouse trials. Our results showed that drought stress significantly affect the plant growth parameters for both studied genotypes.

The effects of the *Rhizobium* strains were quite limited in the plant biomass analysis, even though we observed that OL13 strain led to a significant improvement in the biomass of the MLT genotype, allowing it to approach the biomass values of AQD under drought stress conditions. Since it is known that AQD is recognized as resistant to drought stress [19], these results demonstrate that the resistance of a genotype can be highly dependent on the symbiont strain present. Our data indicated that symbiosis with different rhizobia modified differently physiological and biochemical responses in faba bean under both watering conditions. The Chl and Car content were higher in plants inoculated by 3841 strain compared to OL13 strain, suggesting that the 3841 strain had an efficient effect on photosynthesis. However, the stomatal conductance under limited water condition was similar, regardless of the *Rhizobium* strain. These findings suggest that the mechanism for the control of stomatal conductance is not dependent of the symbiont.

In contrast, considering osmolyte accumulation and antioxidant response, we observed significant differences between the two studied *Rhizobia* strains. Under water-limited conditions, we observed proline accumulation in all studied organs of both studied genotypes as expected from the literature [19,45,46]. Surprisingly, higher content of proline was measured in MLT than in drought resistant AQD genotype, which is described to accumulate this osmolyte more strongly than the Luz d’Otonio (LO) and Reina Mora (RM) genotypes [19]. The accumulation of osmolytes such as proline, soluble sugars or other amino acids is a plant strategy allowing them to regulate the osmotic potential of cells under drought stress [47], and may help to stabilize proteins and cell structures, particularly when the stress becomes severe or persists for longer periods [48]. Despite several studies on proline, the role of proline accumulation is still controversially discussed as it is described to function as a radical scavenger, antioxidant and as involved in the regulation of apoptosis and seed development [49,50]. The most striking point was the effect of OL13 strain inoculation on the level of proline in the three studied organs in the MLT genotype; nodules accumulated notably as much as twice as much proline than MLT nodules with the strain 3841. Bacterial cells generally prevent dehydration by accumulating osmolytes, including proline, which is one of the major osmolytes in rhizobial osmo-adaptation to balance the internal and external water potential [51]. This result could be the consequence of the higher tolerance of the strain OL13 to drought stress; a higher tolerance that could be due to the capacity of OL13 strain to accumulate a higher concentration of proline in stress situations. Moreover, a correlative study demonstrated that the nodules of drought-tolerant soybean cultivar accumulated higher levels of proline compared to the sensitive cultivar [1]. Another striking feature was the fact that *Rhizobium* in nodules can influence the proline concentration in roots and in leaves a mechanism that remains to be elucidated but that has already been observed [52]. Indeed, Irshad et al. [52] observed higher compatible solutes (proline, free amino acids, glycine betaine, soluble sugars, and proteins) on *Medicago truncatula* plant organs with active nodules compare to non-nodulated ones in the salt stress tolerance process. The conclusion of the authors was that *Rhizobium meliloti* inoculation play a key role against salt stress through induction of antioxidant system and accumulation of compatible solutes was advise by the authors [52]. High proline level was also positively correlated with the high level of H_2_O_2_ recorded for MLT genotype. Verslues et al. [53] suggest that H_2_O_2_ can stimulate ABA-induced proline accumulation and may be one factor in inducing the high levels of proline accumulation that can occur under low water potential stress. On the other hand, the high level of H_2_O_2_ recorded in the MLT genotype suggests that this genotype is more drought-sensitive.

Regarding the soluble sugar content, we observed its accumulation in both genotypes. Soluble sugar content was higher in leaves of AQD than in leaves of MLT. Furthermore, under water deficit conditions, a significant increase of soluble sugar content was observed in AQD roots inoculated with 3841 strain. On the contrary, in MLT roots and leaves, higher soluble sugar accumulation was measured in plants inoculated with OL13 strain. Knowing the importance of soluble sugar as an osmolyte and its role in plants’ metabolism and in maintaining plant development under abiotic stress conditions, these findings support the hypothesis that 3841 strain is more beneficial for ADQ whereas OL13 is more beneficial for MLT under water deficit conditions. It should be noted that accumulation of soluble sugars was described as more efficient than proline accumulation to counteract osmotic stress and a putative interaction between proline and soluble sugars has been brought forward [50,54].

Under water-limited condition, both genotypes showed a significant accumulation of MDA and H_2_O_2_ in the two studied organs. However, MDA and H_2_O_2_ accumulation were lower in roots than in shoot for both studied genotypes, showing that the stress was more felt in leaves than in roots. On the other hand, significant lower accumulation of H_2_O_2_ in both organs was detected in AQD compared to MLT. Results again in favor of a higher tolerance of AQD to drought stress. When comparing *Rhizobium* effect, both studied strains showed similar effect on MDA and H_2_O_2_ content. The fact that a lower leaves level of H_2_O_2_ was associated with greater water deficit adaptation suggests that detoxifying mechanisms in the AQD genotype are more efficient than those in the MLT genotype. The activation of the cellular antioxidant machinery is critical for drought-induced oxidative stress protection. In our experiments, SOD, CAT and APX activities were significantly higher in leaves and roots of AQD than in MLT under water deficit. Nevertheless, despite the important increase recorded for GaPX for both genotypes and in all studied organs, this increase was significantly more important in MLT genotype than in AQD genotype. According to Mhadhbi et al. [55,56], GaPX is an important enzyme in the antioxidant defence of nodules under osmotic stress. The induction and protective role of peroxidases under salt and water osmotic stresses, were widely reported in legume-rhizobia nodules [55,57,58,59], and other plant tissues [60,61]. In addition, SOD, CAT, GaPX, and APX activities, were reported to play an important role in symbiosis establishment and nodule protection [62]. In Mhadhbi et al. [56] study, the same enzymes activities were investigated in chickpea–rhizobia associations, and the result showed a high contribution of the rhizobial partner to the total variance of antioxidant activities for all four enzymes as we observed in our work. Similarly, Kumari et al. [63], studies showed that chickpea inoculated by bacterial endophytes presented a significant upregulation of SOD and proline gene expression, indicating that bacterial endophytes may help to protect plants under drought stress conditions by upregulating gene expression at a molecular level.

The *R. leguminosarum* effect was noticed for AQD leaves inoculated with 3841 strain, and MLT leaves inoculated by OL13 strain, showing the greater ability conferred by these strains to improve enzyme activities to scavenge ROS under water-limited conditions. It should be noted that the MLT genotype with 3841 strain accumulates less proline and soluble sugar than with OL13 strain in which a higher catalase and GaPX activity was observed. All together these results suggest that the *Rhizobium* strains affect differently the two studied genotypes by enhancing their antioxidant enzymatic activity to cope with drought stress conditions, results which further show the importance of the *Rhizobium* strain in the plant’s response to water stress. This leads us to suggest that the choice of the two symbiotic partners is critical for achieving greater drought tolerance in varieties which tend to disappear because of their sensitivity and thus preserve a better biodiversity.

## 4. Materials and Methods

### 4.1. Biological Material and Growth Conditions

Two faba bean (*Vicia faba* L. var. major) genotypes, Aquadulce (AQD) and Maltais (MLT), coupled individually with two *Rhizobium leguminosarum* strains were used in this study. The experiment was conducted under greenhouse conditions at the laboratory of Plant Genetics, Biochemistry and Biotechnology (Mentouri Brothers-Constantine I University, Constantine, Algeria), during the winter of 2019 under natural light, air temperature between 22 °C ± 4 °C (night/day), 50 ± 80% of relative humidity and 16 h of photoperiod. Aquadulce (AQD) genotype was received from the National Center of Seeds and Plants Control and Certification (CNCC, Constantine, Algeria), AQD is known to be a tolerant water-deficit faba bean genotype [19]. The second genotype was a local Maltais (MLT) one, which is one the most cultivated genotypes in Algeria.

Before sowing, seeds of the two genotypes were surface sterilized with 70% ethanol for 2 min. After removing the ethanol solution, the seeds were immerged in 5% sodium hypochlorite solution for 10 min, and then the seeds were rinsed three times with sterile deionized water and left to germinate on sterile cotton at 23 °C for six days. Seedlings with homogenous stages of development were selected and sown at 3-cm depth in 4.5 L plastic pots, filled with 3.6 kg potting mix containing air dried sand and peat (4:1, v:v). One seed was sown per 4.5 L pot. The soil moisture was kept at 80% field capacity (FC) in all pots by applying water on alternate days to replace the loss of water due to evaporation until the induction of water deficit treatment.

The two selected rhizobia strains were evaluated in a pot experiment for inducing water deficit stress tolerance in faba bean. The first Rhizobial strain was *Rhizobium leguminosarum bv. viciae* 3841, able to nodulates legumes in the Tribe *Viciae*—*Vicia*, *Pisum*, *Lathyrus*, *Lens*. It was received from the Sophia Agrobiotech Institute (Nice, France). The second rhizobial strain, OL13, was isolated from the nodules of *Lens culinaris* from a semi-arid Algerian region [32]. Rhizobial cells were harvested by centrifugation at 5000× *g* for 20 min (twice 10 min) after culturing in yeast mannitol broth. Then, bacterial cells (pellets) were washed and suspended in sterilized distilled water and uniform bacterial cell density (10^8^ CFU·mL^−1^) was achieved by maintaining the optical density at a wavelength of 600 nm by a spectrophotometer. This suspension of rhizobial cells was used as inoculum. Three days after sowing the faba bean seeds in pots, 10 mL of each inoculum solution was added individually for each plant. Total pots experiment was divided into two parts, whereby half received inoculum 3841 and the other half inoculum OL13.

Water deficit was initiated at 50% flowering stage in half of the pots (40–45 days after sowing), and it was applied by maintaining the soil moisture at 40% of field capacity (FC) for the stressed plants, and 80% FC for the well-watered plants. The experimental pots were arranged in a completely randomized design with twelve replicates per treatment per genotype. Each pot was irrigated with Hoagland-nitrogen free solution [64] once a week from sowing until the end of the experiment. 

### 4.2. Assessment of Physiological and Biochemical Parameters

After 28 days of water deficit (seedling stage), plants were harvested for growth assessment, physiological and biochemical analyses. Measurements were taken between 11:00 and 13:00 local time. Stomatal conductance was measured using a porometer (AP4 Delta-T Devices, Cambridge, UK) on the abaxial surface of the leaflets of the uppermost fully expanded leaf once per plant. 

Chlorophyll and carotenoid contents were measured as described by Takele [65]. 0.2 g of leaf segments from fully expanded third leaves, from each treatment, were extracted in 4 mL of acetone (80%, *v*/*v*) for 48 h at 4 °C. The absorbance of the resulting solution was read by a spectrophotometer at 663, 645 and 470 nm. The total chlorophyll content was determined by using the Lichtenthaler and Wellburn [66] formula. Total chlorophyll was determined using the following formula:

Chlorophyll a: Chl a = 12.21 × DO_663_ − 2.81 × DO_645_ (1)

Chlorophyll b: Chl b = 20.13 × DO_645_ − 5.03 × DO_663_ (2)

Total chlorophyll: Chl = Chl a + Chl b (3)

Total carotenoids (xanthophils + β-carotenes):

Ca = (1000 × DO_470_ − 3.27 × Chl a − 104 × Chl b)/229 (4)

Four replicates per treatment were performed. For dry weight measurements, shoots were removed above the collar region and roots were carefully removed from the soil substrate. Both plant parts were washed and dried in a drying oven at 65 °C until obtaining a constant weight. Six replicates for each treatment per genotype were performed.

### 4.3. Proline and Soluble Sugar Contents 

After 28 days of water deficit, the fully expanded third leaf, roots and nodules were collected for free proline measurements. Free proline was extracted from fresh plant material samples according to the method of Troll and Lindsley [67], as modified by Monneveux and Nemmar [68]. Fresh materials (0.1 g) from each part were extract in 40% methanol for 1 h at 85 °C. Then acetic acid, ninhydrin and orthophosphoric acid were added to 1 mL of the obtained extract and the reaction mixture was incubated at 100 °C in a water bath for 30 min. After cooling the reaction mixtures, 5 mL of toluene were added. After thorough mixing, the chromosphere-containing toluene was aspirated and its absorbance read at 520 nm in spectrophotometer, against a toluene blank. Free proline content in samples was estimated by referring to a standard curve and expressed as μmol proline·g FW^−1^. Three replicates per genotype per treatment were performed.

Soluble sugar content was determined using the method described by Dubois et al. [69]. Fresh materials from each part (shoot and root, 0.1 g) were extracted in 80% ethanol for 48 h and subsequently dried under a hot air stream. The residue was homogenized with 20 mL of water. One milliliter of alcoholic extract was treated with 1 mL of 5% phenol (*v*/*v*) and 5 mL of sulphuric acid. The mixture was kept for 20 min at 30 °C. The soluble sugar content was revealed by measuring the absorbance at 485 nm. Soluble sugar content was estimated by referring to a standard curve, constructed using glucose, and was expressed as mg soluble sugar·g FW^−1^. Three replicates per genotype per treatment were performed.

### 4.4. Lipid Peroxidation and Hydrogen Peroxide Content

After 28 days of water stress, fully expanded third leaves and roots were harvested for oxidative stress indicator measurements. Lipid peroxidation was determined by measuring the amount of malondialdehyde (MDA) produced by the thiobarbituric acid (TBA) reaction as described by Heath and Packer [70]. Leaves (0.1 g) were homogenised in a mortar and pestle with ice-cold 0.1% (*w*/*v*) trichloracetic acid (TCA), and centrifuged at 12,000× *g* for 15 min. The supernatant was used for the determination of MDA content. 0.5 % (*w*/*v*) (TBA) solution containing 20% (*w*/*v*) (TCA) was added to the supernatant. The mixture was heated at 95 °C for 30 min, quickly cooled in an ice-bath, and then centrifuged at 5000× *g* for 5 min for clarification. The MDA concentration was calculated from the absorbance at 532 nm (unspecific turbidity was corrected by subtracting the absorbance at 600 nm) by using an extinction coefficient of 155 mM^−1^. cm^−1^ and the results expressed as nmol MDA. g^−1^ FW. Three replicates per treatment per genotype were performed.

Hydrogen peroxide (H_2_O_2_) was measured as described by Velikova et al. [71]. Fresh leaves (0.1 g) were homogenized in 5 mL of TCA (0.1%) (*w*/*v*). The homogenate was centrifuged at 13,000× *g* for 15 min at 4 °C. The supernatant was then added to 10 mM potassium phosphate buffer (pH 7.0) and 1 M potassium iodide. The absorbance of the supernatant was measured at 390 nm. The H_2_O_2_ content was calculated by its molar extinction coefficient (0.28 µM^−1^·cm^−1^) and the results expressed as nmol H_2_O_2_·g^−1^ FW. Each treatment included three replicates.

### 4.5. Determination of Antioxidant Enzymes Activities

After 28 days of water stress, crude enzyme extracts made from fully expanded third leaves, roots and nodules were used to determine the activities of antioxidant enzymes. Fresh plant tissue from 0.2 g was homogenized in extraction buffer (0.1 mM EDTA, 0.1 % (*v*/*v*) Triton X-100, 1 mM phenylmethanesulfonyl fluoride (PMSF), in potassium phosphate buffer 50 mM, pH 7.8) [45]. The homogenate was centrifuged at 15,000× *g* for 30 min at 4 °C. Supernatant was used for enzyme activity and protein content assays. All extracts were handled at 4 °C. Total protein content of the enzyme extracts was determined following Bradford [72] using bovine serum albumin as a standard. All enzyme activities, except the ·, were expressed as µM × min^−1^·× mg^−1^ of protein. 

Superoxide dismutase (SOD, EC 1.15.11) activity was assayed using the nitroblue tetrazolium (NBT) method of Beauchamp and Fridovich [73]. The assay reaction mixture consisted of 50 mM of phosphate buffer (pH 7.8), 2 mM of EDTA, 9.9 mM of methionine, 55 μM of NBT, 2 μM of riboflavin and enzyme extract. The reaction was initiated by illuminating the samples under a light source for 20 min, the samples were after put in the dark for 20 min. Absorbance of the samples was measured at 560 nm. One unit of SOD activity was defined as the amount of enzyme required to cause 50% inhibition of the reduction of NBT photoreduction at 25 °C. Catalase activity (CAT) (EC 1.11.1.6) was assayed by measuring the initial rate of disappearance of H_2_O_2_ by the method of Chance and Maehly [74]. The decrease in absorbance at 240 nm was measured as a consequence of H_2_O_2_ consumption [75]. In this assay, 25 mM phosphate buffer (pH 7.0) and 30 mM H_2_O_2_ were used in the reaction solution.

The ascorbate peroxidase activity (APX) (EC 1.11.1.11) was assayed using the method of Chen and Asada [76], by measuring the decrease in absorbance at 290 nm caused by ascorbic acid oxidation. In this assay, 50 mM phosphate buffer (pH 7), 0.1 mM EDTA, 5 mM H_2_O_2_ and 0.5 mM sodium ascorbate were used in the reaction solution.

Guaiacol peroxidase (GaPX, EC 1.11.1.7) activity was determined according to Fielding and Hall [77] by the oxidation of guaiacol in the presence of H_2_O_2_ using a reaction solution containing 25 mM phosphate buffer (pH 7.0), 9mM guaiacol, 5 mM H_2_O_2_ and enzyme extract. The increase of absorbance due to formation of tetraguaiacol was assessed at 470 nm [78].

### 4.6. Statistical Analysis

Statistical analysis was performed using XLSTAT Version 2016.02.28451 software (Addinsoft, Paris, France) and two-ways analysis of variance (ANOVA II). The data were expressed as the mean ± standard error. Means were statistically compared using Student-Newman-Keuls’s multiple-range test at the level of *p* < 0.05.

## 5. Conclusions

The present study demonstrated that *R. leguminosarum* could play a role in reducing drought effect, however, the effect of differnt *Rhizobia* strains in alleviating drought stress in the two studied *V. faba* genotypes was different. The results obtained indicate that higher tolerance to drought stress in faba bean genotype is mainly dependent to upregulation of different antioxidative mechanisms and consistent production of compatible solutes which are differentially activated according to the *Rhizobium* nodulating the plant. The MLT variety, widely cultivated in Algeria, proves to have a much better tolerance to water stress in symbiosis with the local *Rhizobium* strain OL13. This OL13 strain leads in particular to a much better production of proline and soluble sugar in MLT. This strain showed also a significant improvement in AQD genotype, but not at the same level and for the same parameters exhibited when inoculated with the 3841 strain. The use of the appropriate *R. leguminosarum* strains appears to be a promising eco-friendly strategy to increase the growth and drought tolerance of faba bean grown in the fore coming era of climate change. However, here is a need to explore more to decipher the mechanism by which a *Rhizobium* strain can affect the drought stress resilience in legumes plants.

## Figures and Tables

**Table 1:** Malondialdehyde contents in leaves and roots.

MDA Content (nmol × g^−1^ FW)					
Organs		Leaves		Roots	
Genotype	Rhiz-treatmnt	80% FC (NS)	40% FC (S)	80% FC (NS)	40% FC (S)
AQD	3841	2.75 ± 0.21 ^d^	4.26 ± 0.28 ^b^	1.68 ± 0.19 ^b^	2.32 ± 0.20 ^a^
	OL13	2.69 ± 0.21 ^d^	3.76 ± 0.23 ^c^	1.65 ± 0.17 ^b^	2.35 ± 0.10 ^a^
MLT	3841	3.08 ± 0.10 ^d^	4.86 ± 0.23 ^a^	1.66 ± 0.16 ^b^	2.69 ± 0.16 ^a^
	OL13	2.97 ± 0.13 ^d^	4.56 ± 0.32 ^ab^	1.61 ± 0.17 ^b^	2.52 ± 0.23 ^a^

Seventy day-old *Vicia faba* plants, previously individually inoculated with *Rhizobium* strains 3841 and OL13, were grown under control conditions (Noted: NS) or under water limited conditions (Noted: S). AQD: Aquadulce; MLT: Maltais. The values shown are the mean ± SD of three independent replicates. The different letters a–d are the groups which are significantly different at the 0.05 level of probability by Student-Newman-Keuls Test (as mentionned in legend).

**Table 2:** Hydrogen peroxide contents in leaves and roots.

H_2_O_2_ Content (nmol × g^−1^ FW)					
Organs		Leaves		Roots	
Genotype	Rhiz.treatmnt	80% FC (NS)	40% FC (S)	80% FC (NS)	40% FC (S)
AQD	3841	32.62 ± 3.22 ^c^	51.19 ± 2.30 ^b^	15.95 ± 1.09 ^c^	20.24 ± 1.49 ^b^
	OL13	29.05 ± 3.38 ^cd^	50.24 ± 0.82 ^b^	14.29 ± 0.71 ^cd^	24.29 ± 3.11 ^b^
MLT	3841	26.43 ± 1.89 ^d^	60.71 ± 3.27 ^a^	15.71 ± 0.71 ^d^	31.90 ± 3.93 ^a^
	OL13	25.48 ± 2.30 ^d^	57.14 ± 1.89 ^a^	15.71 ± 2.47 ^d^	26.43 ± 2.58 ^a^

Seventy day-old *Vicia faba* plants, previously individually inoculated with Rhizobium strains 3841 and OL13, were grown under control conditions (Noted: NS) or under water limited conditions (Noted: S). AQD: Aquadulce; MLT: Maltais. The values shown are the mean ± SD of three independent replicates. The different letters a–d are the groups which are significantly different at the 0.05 level of probability by Student-Newman-Keuls Test (as mentionned in legend).

## Data Availability

The data presented in this study are available in article.

## References

[1] Furlan A.L., Bianucci E., Castro S., Dietz K.J. (2017). Metabolic features involved in drought stress tolerance mechanisms in peanut nodules and their contribution to biological nitrogen fixation. Plant Sci..

[2] Karkanis A., Ntatsi G., Lepse L., Fernández J.A., Vågen I.M., Rewald B., Alsiņa I., Kronberga A., Balliu A., Olle M. (2018). Faba bean cultivation–revealing novel managing practices for more sustainable and competitive European cropping systems. Front. Plant Sci..

[3] Goyal R.K., Mattoo A.K., Schmidt M.A. (2021). Rhizobial–host interactions and symbiotic nitrogen fixation in legume crops toward agriculture sustainability. Front. Microbiol..

[4] Longobardi F., Sacco D., Casiello G., Ventrella A., Sacco A. (2015). Chemical profile of the Carpino broad bean by conventional and innovative physicochemical analyses. J. Food Qual..

[5] Amede T., Schubert S. (2003). Mechanisms of drought resistance in grain II: Stomatal regulation and root growth. SINET Ethiop. J. Sci..

[6] Khan H.R., Paull J.G., Siddique K.H.M., Stoddard F.L. (2010). Faba bean breeding for drought-affected environments: A physiologcal and agronomic perspective. Field Crop. Res..

[7] Khazaei H., Street K., Santanen A., Bari A., Stoddard F.L. (2013). Do faba bean (*Vicia faba* L.) accessions from environments with contrasting seasonal moisture availabilities differ in stomatal characteristics and related traits?. Genet. Resour. Crop. Evol..

[8] Golldack D., Li C., Mohan H., Probst N. (2014). Tolerance to drought and salt stress in plants: Unraveling the signaling networks. Front. Plant Sci..

[9] Hussain M.B., Mahmood S.A.J.I.D., Ahmed N.I.A.Z., Nawaz H. (2018). Rhizobial inoculation for improving growth physiology, nutrition and yield of maize under drought stress conditions. Pak. J. Bot..

[10] Meena K.K., Sorty A.M., Bitla U.M., Choudhary K., Gupta P., Pareek A., Singh D.P., Prabha R., Sahu P.K., Gupta V.K. (2017). Abiotic stress responses and microbe-mediated mitigation in plants: The omics strategies. Front. Plant Sci..

[11] Nadeem M., Li J., Yahya M., Sher A., Ma C., Wang X., Qiu L. (2019). Research progress and perspective on drought stress in le-gumes: A review. Int. J. Mol. Sci..

[12] Maalouf F.M., Mohammed N., Aladdin H., Ahmed A., Xuxiao Z., Shiying B., Tao Y. (2013). Faba bean. Genetic and Genomic Re-Sources of Grain Legume Improvement.

[13] Muktadir M.A., Adhikari K.N., Merchant A., Belachew K.Y., Vandenberg A., Stoddard F.L., Khazaei H. (2020). Physiological and biochemical basis of faba bean breeding for drought adaptation—A review. Agronomy.

[14] Mwanamwenge J., Loss S.P., Siddique K.H.M., Cocks P.S. (1999). Effect of water stress during floral initiation, flowering and podding on the growth and yield of faba bean (*Vicia faba* L.). Eur. J. Agron..

[15] Katerji N., Mastrorilli M., Lahmer F.Z., Maalouf F., Oweis T. (2011). Faba bean productivity in saline–drought conditions. Eur. J. Agron..

[16] Khan H.R., Link W., Hocking T.J., Stoddard F.L. (2007). Evaluation of physiological traits for improving drought tolerance in faba bean (*Vicia faba* L.). Plant Soil.

[17] Alghamdi S.S., Al-Shameri A.M., Migdadi H.M., Ammar M.H., El-Harty E.H., Khan M.A., Farooq M. (2015). Physiological and molecular characterization of faba bean (*Vicia faba* L.) genotypes for adaptation to drought stress. J. Agron. Crop. Sci..

[18] Girma F., Haile D. (2014). Effects of supplemental irrigation on physiological parameters and yield of faba bean (*Vicia faba* L.) varie-ties in the highlands of Bale, Ethiopia. J. Agron..

[19] Kabbadj A., Makoudi B., Mouradi M., Pauly N., Frendo P., Ghoulam C. (2017). Physiological and biochemical responses involved in water deficit tolerance of nitrogen-fixing *Vicia faba*. PLoS ONE.

[20] Mansour E., Desoky E.S.M., Ali M.M., Abdul-Hamid M.I., Ullah H., Attia A., Datta A. (2021). Identifying drought-tolerant genotypes of faba bean and their agro-physiological responses to different water regimes in an arid Mediterranean environment. Agric. Water Manag..

[21] Hayatu M., Muhammad S.Y., Abdu H.U. (2014). Effect of water stress on the leaf relative water content and yield of some cow-pea (*Vigna unguiculata* (L) *Walp.*) genotype. Int. J. Sci. Technol. Res..

[22] Benjamin J.G., Nielsen D.C., Vigil M.F., Mikha M.M., Calderon F. (2014). Water deficit stress effects on corn (*Zea mays*, L.) root: Shoot ratio. Open J. Soil Sci..

[23] Xiang D.B., Peng L.X., Zhao J.L., Zou L., Zhao G., Song C. (2013). Effect of drought stress on yield, chlorophyll contents and photosynthesis in tartary buckwheat (*Fagopyrum tataricum*). J. Food Agric. Environ..

[24] Pandey V., Shukla A. (2015). Acclimation and tolerance strategies of rice under drought stress. Rice Sci..

[25] Nasr Esfahani M., Sulieman S., Schulze J., Yamaguchi-Shinozaki K., Shinozaki K., Tran L.S.P. (2014). Mechanisms of physiological adjustment of N2 fixation in *Cicer arietinum* L. (chickpea) during early stages of water deficit: Single or multi-factor controls. Plant J..

[26] Dhanushkodi R., Matthew C., McManus M.T., Dijkwel P.P. (2018). Drought-induced senescence of *Medicago truncatula* nodules involves serpin and ferritin to control proteolytic activity and iron levels. New Phytol..

[27] Mohammadkhani N., Heidari R. (2008). Effects of drought stress on soluble proteins in two maize varieties. Turk. J. Biol..

[28] Siddiqui M.H., Khan M.N., Mohammad F., Khan M.M.A. (2008). Role of nitrogen and gibberellin (GA3) in the regulation of en-zyme activities and in osmoprotectant accumulation in *Brassica juncea* L. under salt stress. J. Agron. Crop Sci..

[29] Raja V., Majeed U., Kang H., Andrabi K.I., John R. (2017). Abiotic stress: Interplay between ROS, hormones and MAPKs. Environ. Exp. Bot..

[30] Foyer C.H., Noctor G. (2016). Stress-triggered redox signalling: What’s in pROSpect?. Plant Cell Environ..

[31] Mittler R. (2017). ROS are good. Trends Plant Sci..

[32] Riah N. (2015). Diversité et Structure Génétique des Populations de *Rhizobium leguminosarum* Symbiovar Viciae Isolées du Pois (*Pisum sativum*) et de la Lentille (*Lens culinaris*) Cultivés dans Deux Zones Éco-Climatiques Subhumide et Semi-Aride de l’est Algérien. Ph.D. Thesis.

[33] El-Tayeb M., Hassanein A.M. (2000). Germination, seedling growth, some organic solutes and peroxidase expression of different *Vicia faba* lines as influenced by water sterss. Acta Agron. Hung..

[34] Le Thiec D., Manninen S. (2003). Ozone and water deficit reduced growth of Aleppo pine seedlings. Plant Physiol. Biochem..

[35] Devi S.P.S., Sujatha B. (2014). Drought-induced accumulation of soluble sugars and proline in two pigeon pea (*Cajanus Cajan* L. Millsp.) cultivars. Int. J. Innov. Res. Dev..

[36] Gontia-Mishra I., Sapre S., Sharma A., Tiwari S. (2016). Amelioration of drought tolerance in wheat by the interaction of plant growth-promoting rhizobacteria. Plant Biol..

[37] Sgherri C.L.M., Maffei M., Navari-Izzo F. (2000). Antioxidative enzymes in wheat subjected to increasing water deficit and rewa-tering. J. Plant Physiol..

[38] Apel K., Hirt H. (2004). Reactive oxygen species: Metabolism, oxidative stress, and signal transduction. Annu. Rev. Plant Biol..

[39] Miller G., Suzuki N., Ciftci-Yilmaz S., Mittler R. (2010). Reactive oxygen species homeostasis and signalling during drought and salinity stresses. Plant Cell Environ..

[40] Rasool S., Ahmad A., Siddiqi T.O., Ahmad P. (2013). Changes in growth, lipid peroxidation and some key antioxidant enzymes in chickpea genotypes under salt stress. Acta Physiol. Plant..

[41] Chiboub M., Jebara S.H., Abid G., Jebara M. (2020). Co-inoculation effects of *Rhizobium sullae* and *Pseudomonas* sp. on growth, antioxidant status, and expression pattern of genes associated with heavy metal tolerance and accumulation of cadmium in Sulla co-ronaria. J. Plant Growth Regul..

[42] Wang Y., Zhang Z., Zhang P., Cao Y., Hu T., Yang P. (2016). Rhizobium symbiosis contribution to short-term salt stress tolerance in alfalfa (*Medicago sativa* L.). Plant Soil.

[43] Ferguson B.J., Indrasumunar A., Hayashi S., Lin M.H., Lin Y.H., Reid D.E., Gresshoff P.M. (2010). Molecular analysis of legume nodule development and autoregulation. J. Integr. Plant Biol..

[44] Dwivedi S.L., Sahrawat K.L., Upadhyaya H.D., Mengoni A., Galardini M., Bazzicalupo M., Biondi E.G., Hungria M., Kaschuk G., Blair M.W. (2015). Advances in host plant and rhizobium genomics to enhance symbiotic nitrogen fixation in grain legumes. Advances in Agronomy.

[45] Abid G., M’hamdi M., Mingeot D., Aouida M., Aroua I., Muhovski Y., Sassi K., Souissi F., Mannai K., Jebara M. (2017). Effect of drought stress on chlorophyll fluorescence, antioxidant enzyme activities and gene expression patterns in faba bean (*Vicia faba* L.). Arch. Agron. Soil Sci..

[46] Khan N., Bano A., Rahman M.A., Guo J., Kang Z., Babar M. (2019). Comparative physiological and metabolic analysis reveals a complex mechanism involved in drought tolerance in chickpea (*Cicer arietinum* L.) induced by PGPR and PGRs. Sci. Rep..

[47] Zhang Y., Zhong C.L., Chen Y., Chen Z., Jiang Q.B., Wu C., Pinyopusarerk K. (2010). Improving drought tolerance of *Casuarina equisetifolia* seedlings by arbuscular mycorrhizas under glasshouse conditions. New For..

[48] Camaille M., Fabre N., Clément C., Ait Barka E. (2021). Advances in wheat physiology in response to drought and the role of plant growth promoting rhizobacteria to trigger drought tolerance. Microorganisms.

[49] Kocsy G., Laurie R., Szalai G., Szilágyi V., Simon-Sarkadi L., Galiba G., De Ronde J.A. (2005). Genetic manipulation of proline le-vels affects antioxidants in soybean subjected to simultaneous drought and heat stresses. Physiol. Plant..

[50] Kavi Kishor P.B., Sreenivasulu N. (2014). Is proline accumulation per se correlated with stress tolerance or is proline homeostasis a more critical issue?. Plant Cell Environ..

[51] Kibido T., Kunert K., Makgopa M., Greve M., Vorster J. (2020). Improvement of rhizobium-soybean symbiosis and nitrogen fixation under drought. Food Energy Secur..

[52] Irshad A., Rehman R.N.U., Abrar M.M., Saeed Q., Sharif R., Hu T. (2021). Contribution of rhizobium–legume symbiosis in salt stress tolerance in medicago truncatula evaluated through photosynthesis, antioxidant enzymes, and compatible solutes accumulation. Sustainability.

[53] Verslues P.E., Kim Y.S., Zhu J.K. (2007). Altered ABA, proline and hydrogen peroxide in an Arabidopsis glutamate: Glyoxylate aminotransferase mutant. Plant Mol. Biol..

[54] Gurrieri L., Merico M., Trost P., Forlani G., Sparla F. (2020). Impact of drought on soluble sugars and free proline content in selected Arabidopsis mutants. Biology.

[55] Mhadhbi H., Jebara M., Limam F., Aouani M.E. (2004). Rhizobial strain involvement in plant growth, nodule protein composition and antioxidant enzyme activities of chickpea-rhizobia symbioses: Modulation by salt stress. Plant Physiol. Biochem..

[56] Mhadhbi H., Jebara M., Zitoun A., Limam F., Aouani M.E. (2008). Symbiotic effectiveness and response to mannitol-mediated osmotic stress of various chickpea–rhizobia associations. World J. Microbiol. Biotechnol..

[57] Jebara S., Jebara M., Limam F., Aouani M.E. (2005). Changes in ascorbate peroxidase, catalase, guaiacol peroxidase and superoxide dismutase activities in common bean (*Phaseolus vulgaris*) nodules under salt stress. J. Plant Physiol..

[58] Nandwal A.S., Kukreja S., Kumar N., Sharma P.K., Jain M., Mann A., Singh S. (2007). Plant water status, ethylene evolution, N2-fixing efficiency, antioxidant activity and lipid peroxidation in *Cicer arietinum* L. nodules as affected by short-term salinization and desalinization. J. Plant Physiol..

[59] Mhadhbi H., Fotopoulos V., Djebali N., Polidoros A.N., Aouani M.E. (2009). Behaviours of Medicago truncatula–Sinorhizobium meliloti symbioses under osmotic stress in relation with the symbiotic partner input: Effects on nodule functioning and protection. J. Agron. Crop Sci..

[60] Türkan I., Bor M., Özdemir F., Koca H. (2005). Differential responses of lipid peroxidation and antioxidants in the leaves of drought-tolerant *P. acutifolius* Gray and drought-sensitive *P. vulgaris* L. subjected to polyethylene glycol mediated water stress. Plant Sci..

[61] Zhang Y.P., Nan Z.B. (2007). Growth and anti-oxidative systems changes in Elymus dahuricus is affected by Neotyphodium endophyte under contrasting water availability. J. Agron. Crop Sci..

[62] Matamoros M.A., Dalton D.A., Ramos J., Clemente M.R., Rubio M.C., Becana M. (2003). Biochemistry and molecular biology of antioxidants in the rhizobia-legume symbiosis. Plant Physiol..

[63] Kumari V., Germida J., Vujanovic V. (2018). Legume endosymbionts: Drought stress tolerance in second-generation chickpea (*Cicer arietinum*) seeds. J. Agron. Crop Sci..

[64] Hoagland D.R., Arnon D.I. (1938). The Water-Culture Method for Growing Plants without Soil.

[65] Takele A. (2010). Differential responses of electrolyte leakage and pigment compositions in maize and sorghum after exposure to and recovery from pre-and post-flowering dehydration. Agric. Sci. China.

[66] Lichtenthaler H.K., Wellburn A.R. (1983). Determinations of total carotenoids and chlorophylls a and b of leaf extracts in different solvents. Biochem. Soc. Trans..

[67] Troll W., Lindsley J. (1955). A photometric method for the determination of proline. J. Biol. Chem..

[68] Monneveux P., Nemmar M. (1986). Contribution à l’étude de la résistance à la sécheresse chez le blé tendre (*Triticum aestivum* L.) et chez le blé dur (*Triticum durum Desf*.): Étude de l’accumulation de la proline au cours du cycle de développement. Agronomie.

[69] Dubois M., Gilles K.A., Hamilton J.K., Rebers P.T., Smith F. (1956). Colorimetric method for determination of sugars and related substances. Anal. Chem..

[70] Heath R.L., Packer L. (1968). Photoperoxidation in isolated chloroplasts: I. Kinetics and stoichiometry of fatty acid peroxidation. Arch. Biochem. Biophys..

[71] Velikova V., Yordanov I., Edreva A. (2000). Oxidative stress and some antioxidant systems in acid rain-treated bean plants: Protective role of exogenous polyamines. Plant Sci..

[72] Bradford M.M. (1976). A rapid and sensitive method for the quantitation of microgram quantities of protein utilizing the principle of protein-dye binding. Anal. Biochem..

[73] Beauchamp C., Fridovich I. (1971). Superoxide dismutase: Improved assays and an assay applicable to acrylamide gels. Anal. Biochem..

[74] Chance B., Maehly A.C. (1955). The assay of catalases and peroxidases. Methods Biochem. Anal..

[75] Havir E.A., McHale N.A. (1987). Biochemical and developmental characterization of multiple forms of catalase in tobacco leaves. Plant Physiol..

[76] Chen G.X., Asada K. (1992). Inactivation of ascorbate peroxidase by thiols requires hydrogen peroxide. Plant Cell Physiol..

[77] Fielding J.L., Hall J.L. (1978). A biochemical and cytochemical study of peroxidase activity in roots of *Pisum sativum*: II. distribution of enzymes in relation to root development. J. Exp. Bot..

[78] Klapheck S., Zimmer I., Cosse H. (1990). Scavenging of hydrogen peroxide in the endosperm of *Ricinus communis* by ascorbate peroxidase. Plant Cell Physiol..

